# Diffusion Magnetic Resonance Imaging Models for Detecting Brain Microstructural Abnormalities in Type 2 Diabetes: A Systematic Review

**DOI:** 10.3390/bioengineering13070730

**Published:** 2026-06-24

**Authors:** Yahui You, Juan Wang, Yongli Yan, Shuoqi Zhang, Wenzhen Zhu, Ying Xiong

**Affiliations:** 1 Department of Radiology, Tongji Hospital, Tongji Medical College, Huazhong University of Science and Technology, 1095 Jiefang Ave, Wuhan 430030, China; yyahui0226@163.com (Y.Y.);; 2 Department of Endocrinology, Tongji Hospital, Tongji Medical College, Huazhong University of Science and Technology, Wuhan 430030, China

**Keywords:** type 2 diabetes mellitus, diffusion magnetic resonance imaging, cognitive impairment

## Abstract

The global prevalence of type 2 diabetes mellitus (T2DM) has increased more than twofold over the last thirty years. T2DM is associated with multiple complications, among which diabetic encephalopathy and accompanying cognitive impairment have drawn considerable interest. This systematic review synthesizes findings from advanced diffusion magnetic resonance imaging (dMRI) studies (published from 2009 to 2025) on T2DM-related brain microstructural abnormalities. The most common technique, diffusion tensor imaging (DTI), consistently reveals reduced white-matter integrity (lower fractional anisotropy, higher diffusivity) associated with cognitive impairment. DTI-based network analysis further identifies disrupted structural network topology, characterized by reduced global and nodal efficiency. To overcome DTI’s limitations, newer techniques provide more specific insights: diffusion kurtosis imaging shows reduced tissue complexity in white matter, gray matter, and crossing-fiber regions via non-Gaussian modeling; neurite orientation dispersion and density imaging quantifies decreased neurite density; intravoxel incoherent motion assesses combined microstructural and microvascular alterations; diffusion spectrum imaging maps complex fiber architecture. These dMRI metrics may provide promising imaging markers for characterizing T2DM-related brain microstructural alterations. However, most available evidence remains cross-sectional, and further longitudinal, multicenter validation is required before these measures can be considered clinically validated biomarkers for prediction, diagnosis, or monitoring.

## 1. Introduction

Type 2 diabetes mellitus (T2DM) is a chronic metabolic disorder characterized by insulin resistance and persistent hyperglycemia. It has become a major global health issue as its prevalence has increased significantly in recent years [1,2]. In addition to its established detrimental effects on the cardiovascular system, kidneys, retina, and feet, patients with T2DM frequently experience cerebral microstructural abnormalities and cognitive impairment [3]. These changes are linked to diabetes-related metabolic disorders, cerebrovascular disease, and neuroinflammation. Diabetes-associated cognitive dysfunction (DACD) is clinically characterized by impairments in executive function, visuospatial abilities, memory, attention, and information processing speed. Despite numerous studies on the subject, the specific pathophysiological mechanisms underlying DACD are not fully elucidated.

Diffusion magnetic resonance imaging (dMRI) and several advanced models are effective non-invasive neuroimaging techniques that provide novel insights into brain architecture. By quantifying water molecule diffusion, these techniques enable the characterization of tissue anisotropy and microstructural integrity. Diffusion tensor imaging (DTI) quantifies water diffusion anisotropy to evaluate white-matter (WM) fiber bundle integrity and orientation [4]. Pathological alterations including demyelination and axonal damage modify water diffusion patterns, which are captured through DTI parametric changes [5]. A key limitation of DTI, however, is its assumption of Gaussian water diffusion, which oversimplifies the complex brain tissue environment. To overcome these limitations, new models have been developed to address these limitations, such as diffusion kurtosis imaging (DKI), neurite orientation dispersion and density imaging (NODDI), intravoxel incoherent motion (IVIM), free-water imaging (FWI), and diffusion spectrum imaging (DSI). These models may provide increased sensitivity to subtle microstructural alterations associated with T2DM. However, whether such alterations can be used for early diagnosis, risk stratification, or clinical decision-making remains uncertain and requires further longitudinal validation. This review provides an overview of dMRI application for detecting brain microstructural changes in T2DM, emphasizing the implications of the results and the association with DACD.

## 2. Materials and Methods

### 2.1. Study Design

This study was conducted as a systematic review with narrative synthesis to summarize the current evidence on dMRI models for detecting brain microstructural abnormalities in T2DM. The review process followed the Preferred Reporting Items for Systematic Reviews and Meta-Analyses (PRISMA 2020) statement [6]. As substantial heterogeneity was anticipated across studies in participant characteristics, diffusion MRI models, acquisition parameters, image-processing methods, and reported outcomes, a quantitative meta-analysis was not considered appropriate. This review was not registered.

### 2.2. Search Strategy

A comprehensive literature search was conducted in PubMed and Web of Science Core Collection to identify studies investigating brain microstructural abnormalities in patients with T2DM using dMRI. The search covered publications from 30 December 2009 to 3 October 2025 and was limited to studies published in English. In addition to electronic database searching, the reference lists of all eligible articles and relevant review papers were manually screened to identify additional potentially relevant studies.

The PubMed search strategy was as follows:

((“Diabetes Mellitus, Type 2”[Mesh] OR “type 2 diabetes mellitus”[Title/Abstract] OR T2DM[Title/Abstract]) AND (“diffusion magnetic resonance imaging”[Title/Abstract] OR “diffusion MRI”[Title/Abstract] OR “diffusion weighted imaging”[Title/Abstract] OR “diffusion tensor imaging”[Title/Abstract] OR DTI[Title/Abstract] OR “diffusion kurtosis imaging”[Title/Abstract] OR DKI[Title/Abstract] OR “neurite orientation dispersion and density imaging”[Title/Abstract] OR NODDI[Title/Abstract] OR “intravoxel incoherent motion”[Title/Abstract] OR IVIM[Title/Abstract] OR “diffusion spectrum imaging”[Title/Abstract] OR DSI[Title/Abstract] OR “free-water imaging”[Title/Abstract] OR FWI[Title/Abstract])).

The Web of Science Core Collection search strategy was as follows:

((“type 2 diabetes mellitus” OR T2DM) AND (“diffusion magnetic resonance imaging” OR “diffusion MRI” OR “diffusion weighted imaging” OR “diffusion tensor imaging” OR DTI OR “diffusion kurtosis imaging” OR DKI OR “neurite orientation dispersion and density imaging” OR NODDI OR “intravoxel incoherent motion” OR IVIM OR “diffusion spectrum imaging” OR DSI OR “free-water imaging” OR FWI)).

### 2.3. Eligibility Criteria

Studies were included if they met the following criteria:

(1) Involved human participants with T2DM;
(2) Applied diffusion MRI techniques to assess brain microstructural abnormalities;
(3) Reported original research data;
(4) Included imaging findings derived from DTI, DTI-based structural network analysis, DKI, NODDI, IVIM, DSI, FWI, or related dMRI approaches;
(5) Were published in English.

Studies were excluded if they were:

(1) Animal studies, reviews, systematic reviews, meta-analyses, or case reports;
(2) Not focused on brain diffusion MRI findings in T2DM.

### 2.4. Study Selection

**Figure 1:**
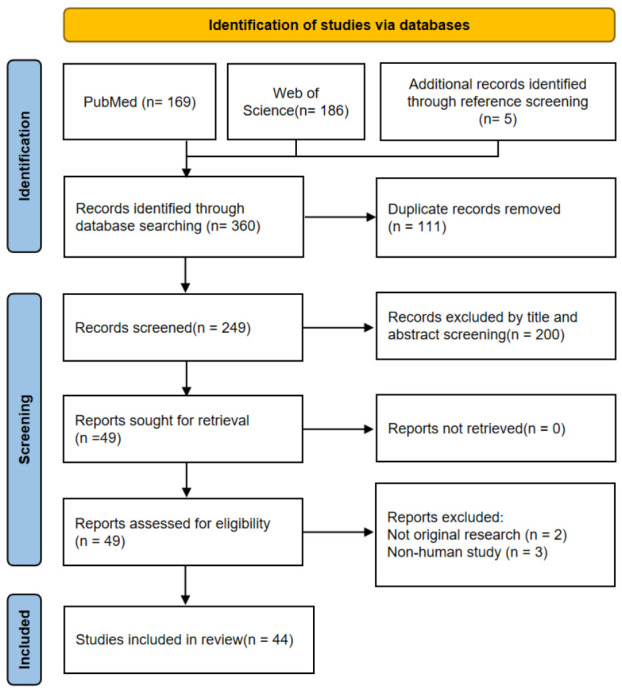
Flow diagram of literature search, screening, eligibility assessment, and final study inclusion.

A total of 355 records were identified through database searching (PubMed, *n* = 169; Web of Science Core Collection, *n* = 186), and an additional five records were identified through reference screening. After removing 111 duplicate records, 249 records remained for title and abstract screening. Of these, 200 records were excluded. Forty-nine full-text articles were assessed for eligibility. Five reports were excluded, including two non-original research articles and three non-human studies. Ultimately, 44 studies were included in the systematic review (Figure 1). The screening process was conducted independently by two reviewers. Extracted information included the first author, year of publication, study population, participant characteristics, diffusion MRI technique, acquisition and analysis methods, investigated brain regions or networks, diffusion-derived metrics, cognitive or clinical assessments, and main findings. Disagreements were resolved through discussion and, when necessary, consultation with a third reviewer.

### 2.5. Quality Assessment

The methodological quality of the included studies was assessed using a modified quality assessment framework for diffusion MRI studies, covering participant selection, control-group matching, MRI acquisition reporting, preprocessing transparency, confounder adjustment, multiple-comparison correction, and reporting of cognitive or clinical outcomes. Each domain was scored from 0 to 1, yielding a total score of 0–7. Studies were classified as high (≥6), moderate (5–5.5), or low quality (<5). Detailed results are provided in Supplementary Table S1.

### 2.6. Data Synthesis

Given the heterogeneity in participant characteristics, imaging models, acquisition protocols, and reported outcomes, findings were synthesized narratively and organized according to dMRI technique, including DTI, DTI-based structural network analysis, DKI, NODDI, IVIM, DSI, and FWI.

## 3. Diffusion MRI Techniques

DTI applies diffusion gradients in multiple directions to measure water molecule diffusion. Fractional anisotropy (FA), mean diffusivity (MD), axial diffusivity (Da), and radial diffusivity (Dr) are the primary parameters. FA quantifies the degree of diffusion anisotropy; MD measures the mean diffusivity of water molecules; Da and Dr correspond to diffusion parallel and perpendicular to the axonal direction, respectively [7]. These parameters are used to assess the integrity of axons, myelin sheaths [8], and the WM microstructure. However, DTI overlooks the non-Gaussian properties of biological tissues and cannot identify the microstructural changes in gray matter, which is primarily constituted of neuronal somata [9]. In some pathological conditions, these parameters may yield values similar to those of normal tissue [10].

**Figure 2:**
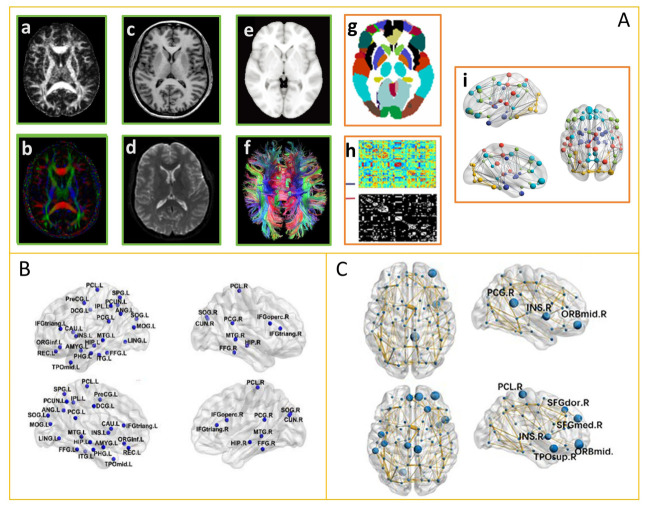
The flowchart of the brain WM network constructed by DTI. Rigid coregistration aligns individual T1-weighted images (**c**) to (**a**)/(**b**), then coregistered to (**d**). Nonlinear registration from the T1 image in native space to (**e**). WM fibers (**f**) are reconstructed, and inverse transformation projects (**g**) to native DTI space for subject-specific parcellation. Weighted/binary and WM structural networks are shown in (**h**)/(**i**) (**A**). Blue dots indicate a significant reduction in nodal efficiency in patients with T2DM comorbid with mild cognitive impairment (MCI) compared to the controls (**B**). Blue dots indicate decreased nodal efficiency in cognitively normal T2DM patients (**top**) and T2DM-MCI patients (**bottom**) versus controls (**C**). Node size indicates the significance of differences between groups. Panels (**A**) and (**C**) were adapted with permission from Xiong et al. [14]. Panel (**B**) was adapted from Li et al. [15] distributed under the terms of the Creative Commons Attribution License (CC BY).

The DTI-based structural network models WM architecture and connectivity, reconstructed from DTI data using graph theory [11] (Figure 2A). Brain regions serve as nodes and fiber-tract connections between these regions are defined as edges [12]. Topological parameters such as clustering coefficient, characteristic path length (Lp), global efficiency, and local efficiency quantify network organization [13]. The clustering coefficient indicates the extent of grouping among network nodes. The Lp denotes the average minimum distance between nodes. The global efficiency quantifies the network’s entire capacity for information transmission, and local efficiency indicates the information processing capacity of a specific region inside the network.

DKI models the non-Gaussian characteristics of water molecule diffusion and quantifies key parameters including mean kurtosis (MK), axial kurtosis (Ka), and radial kurtosis, as well as DTI parameters [16], thereby offering a more thorough understanding of tissue complexity. DKI can be utilized to evaluate changes both in white and gray matter [17], and to evaluate WM neurodegeneration in intricate architectures, including voxels with crossing fibers [18]. DKI also has several limitations, including a longer acquisition time, more sensitivity to noise and artifacts and greater complexity compared to DTI. Unlike DTI, DKI requires a minimum of 2 nonzero b-values (higher b-value typically ≥2000 s/mm^2^) and 15 diffusion directions. Consequently, careful consideration of study design is essential in DKI research because substantial variability may exist in data acquisition and analysis.

NODDI enables advanced characterization of neural microenvironments by modeling water diffusion within axons, dendrites, and glial cells. According to this multi-compartment biophysical model, each voxel is partitioned into three distinct compartments: intracellular, extracellular, and cerebrospinal fluid. Water diffusion is modeled across three distinct compartments: the intracellular space, characterized by restricted diffusion modeled as sticks (Watson distribution); the extracellular space, characterized by hindered diffusion (an anisotropic Gaussian distribution); and the cerebrospinal fluid compartment, characterized by isotropic Gaussian diffusion [19]. The intracellular volume fraction (ICVF, representing neuronal synapse density), orientation dispersion index (reflecting the degree of dispersion of neuronal synapse orientations), and isotropic volume fraction (representing extracellular isotropic free-water volume fraction) are obtained from the calculations, which more directly reflect the changes in brain microstructure. These indices demonstrate significant correspondence with histology data in both animal and human studies [20,21]. Moreover, akin to DKI, NODDI necessitates an extended acquisition duration, while ICVF and isotropic volume fraction mandate a minimum of two shell diffusion datasets [22]. NODDI does not have any direct diffusivity estimates; presupposing equal intracellular and extracellular diffusivity may result in less information about the microstructure. Any deviation from these fixed values will bias the rest of the parameters, reducing their specificity [18].

IVIM can concurrently evaluate alterations in microcirculation and extracellular matrix by distinguishing between diffusion and perfusion components [23,24]. Its principle is based on the diffusion of water molecules and the phenomenon of microcirculatory perfusion. The signal attenuation is decomposed into the parameters of diffusion coefficient, perfusion fraction (f), and pseudo-diffusion coefficient through multi-b-value diffusion-weighted imaging. The diffusion coefficient value mainly reflects the diffusion characteristics of water molecules, which are correlated with the integrity of neural tissues; the f-value indicates the proportion of water molecules related to perfusion, which can reflect the blood volume and microcirculatory perfusion; and the pseudo-diffusion coefficient value reflects the movement of water molecules in the microvessels. These metrics describe cerebral microstructure and microperfusion from complementary biophysical perspectives, enabling multidimensional assessment of alterations in T2DM.

DSI, a high-angular resolution diffusion MRI technique, enables reconstruction of complex WM architectures including axonal crossing and branching fiber architectures [25,26]. The diffusion signal is obtained by applying diffusion gradients of different directions and intensities (higher b-values), which are converted into a diffusion probability density function by Fourier transform. The probability density function delineates the probability of water molecule diffusion in various directions, with its form indicating the orientation and intricacy of the fiber bundles. Furthermore, DSI can extract parameters such as the generalized fractional anisotropy (GFA) and the fiber orientation distribution function.

FWI models the diffusion signal as originating from two distinct compartments: the free water compartment (e.g., cerebrospinal fluid), and the tissue compartment (e.g., white/gray matter). The diffusion signal is decomposed into free-water and tissue-specific components, so as to eliminate the interference of free water on the diffusion signal and evaluate the microstructure of brain more accurately. By analyzing the diffusion signal, the free water volume fraction and the diffusion parameters of tissue water (including FA, MD, etc.) are derived to more precisely represent the microstructural properties of the brain. A comparative overview of these diffusion MRI models, including their main metrics, sensitivity, clinical feasibility, and evidence maturity in T2DM, is provided in Table 1.

## 4. Application in T2DM

### 4.1. Application of DTI in T2DM

#### 4.1.1. Regional White-Matter Abnormalities

Patients with T2DM often exhibit WM abnormalities in the frontal, temporal, and parietal lobes. Many investigations have identified elevated MD and Dr/Da in certain frontal and temporal WM fiber bundles, indicating potential fiber injury [27,28]. Hsu et al. found a decrease in FA in bilateral frontal WM, caused by increased radial diffusivity, suggesting possible demyelination [29]. Another study found that patients showed significantly decreased FA and increased MD and Da in several WM regions associated with the default mode network [30]. Areas of FA reduction included the right frontal lobe, bilateral parietal lobes, and the right middle temporal gyrus. These cerebral areas are crucial to cognition, supporting executive functions, decision-making, attention, memory, language activities and spatial cognition. WM microstructural changes may disrupt inter-regional neuronal transmission, thus impairing cognitive performance.

**Figure 3:**
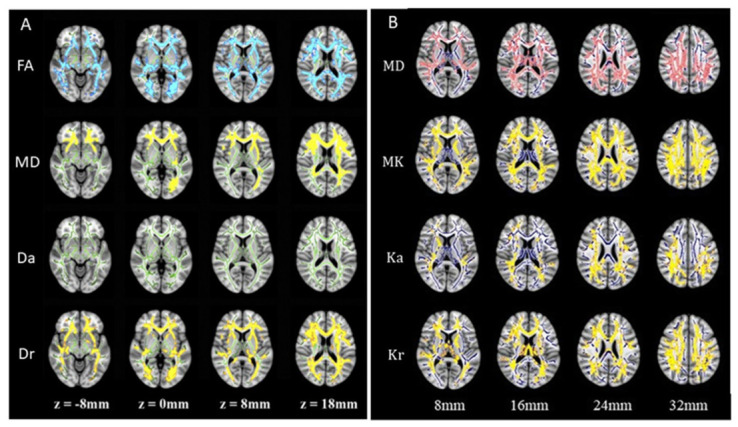
DTI and DKI parameters differ significantly between T2DM and control groups. Tract-based spatial statistics (TBSS) studies revealed reduced FA (blue) and elevated MD/Dr (red-yellow) (*p* < 0.05, FWE-corrected). The green color represents the mean FA skeleton (**A**). Differences in diffusion tensor metrics (increased MD, pink) and kurtosis metrics (decreased MK, Ka and Kr, yellow) between T2DM and control groups using a TBSS analysis (**B**). Panel (**A**) was adapted with permission from Zhang et al. [35]. Panel (**B**) was adapted with permission from Xiong et al. [39].

The corpus callosum (CC) facilitates interhemispheric communication. Diabetes-induced degradation impairs this integration. The cingulate gyrus is strongly associated with the limbic system and engaged in emotion, memory, etc. DTI revealed WM anomalies, notably in CC [31,32,33,34,35,36]. Liu et al. found that T2DM patients had higher MD, Da, and Dr values in the genu and splenium of CC [37]. The automated fiber quantification method showed that T2DM patients had abnormal WM fiber tracts including the right superior longitudinal fasciculus (decreased FA), left corticospinal tract (increased MD), and bilateral fronto-occipital fasciculus [38]. Diffusion tensor tractography revealed decreased FA in bilateral cingulum bundle, increased MD in the right side and shorter fiber length in the left side (Figure 3A). These fiber bundle abnormalities impact interhemispheric information exchange and limbic system connectivity, adversely affecting cognitive and emotional functions.

As key WM tracts, the internal capsule (major projection fibers), external capsule (frontotemporal connections), and corona radiata (cortical-subcortical pathways) support critical functions. Patients with T2DM exhibited impaired WM integrity in these areas [40], as indicated by reduced FA and elevated MD. The anterior and posterior limbs of the internal capsule, the external capsule, and various regions of the corona radiata were impacted [35]. Microstructural alterations in these regions may interfere with the transmission of neural signals between the cerebral cortex and subcortical structures, affecting motor, sensory and cognitive functions.

The thalamus is an important relay station that has extensive connections with several brain regions. Research indicated that in the thalamus concerning the fornix, anterior thalamic radiation, and posterior thalamic radiation, patients with T2DM had decreased FA values and elevated MD and Da values [30,41]. Roy et al. demonstrated that thalamic MD values were modified and linked with cognitive scores. Such changes may disrupt thalamocortical information flow, causing brain network dysfunction [42].

Fang et al. found that anatomical connections of the cerebellum and cerebellar-cerebellar circuits were reduced in patients with T2DM [43]. Additional investigations corroborated that the MD of the cerebellum increases [29], whereas FA decreases [30]. This indicated that diabetes could influence the cerebellum’s function in motor coordination and cognitive control (Table 2).

Overall, the findings summarized in Table 2 demonstrate a relatively consistent pattern of reduced FA and increased diffusivity across major white-matter pathways in T2DM. The most frequently affected regions include the corpus callosum, cingulum bundle, corona radiata, internal capsule, and long-range association fibers. These abnormalities suggest widespread disruption of white-matter integrity and support the hypothesis that T2DM is associated with diffuse microstructural brain injury rather than isolated regional changes.

#### 4.1.2. Associations with Cognitive Function

Cognitive dysfunction in T2DM encompasses various domains, such as memory, information processing speed, executive functioning, and attention. Cognitive deficiencies correlated with diminished WM microstructural integrity in the frontal, temporal, and parietal lobes of the brain [56]. Yau et al. [44] found that FA in the left temporal stem was positively correlated with immediate declarative memory. This finding suggests that the microstructural integrity of the WM in the left temporal lobe is important for memory performance. The more severe WM damage in the anterior limb of the internal capsule and the left external capsule, the more severe executive dysfunction is, according to Zhang et al. [35].

Liu et al. demonstrated that the D value of the right inferior longitudinal fasciculus was an independent predictor of Montreal Cognitive Assessment (MoCA) visuospatial score, suggesting that WM injury in this region impairs visuospatial function [37]. Yu et al. demonstrated a positive correlation between the diffusion tensor image analysis along the perivascular space index and the MoCA score in patients with T2DM. These patients exhibited increased MD and decreased FA values, showing that alterations in WM microstructure are connected to cognitive decline [57].

Disruptions of connections between brain regions contribute to cognitive decline. Decreased WM connections between the hippocampus and the frontal lobe may hinder information transfer and integration, resulting in compromised cognitive function [48]. Hoogenboom et al. discovered that FA values of the cingulum bundle were correlated with the strength of functional connectivity between the posterior cingulate and the medial frontal gyrus within the default mode network, and that changes in this functional connectivity were linked to cognitive decline [46].

Similar WM abnormalities were observed in patients with T2DM and other cognitive disorders. Studies have identified overlapping alterations in brain regions affected in patients with T2DM and Alzheimer’s disease. Tan et al. demonstrated that patients with T2DM exhibited WM abnormalities in regions associated with the default mode network, which were also frequently affected in patients with Alzheimer’s disease [30]. A further study indicated that patients with both Alzheimer’s disease and T2DM exhibited considerable dysfunction in the left superior longitudinal fasciculus, along with increased creatinine levels [58]. This finding suggested that T2DM may increase the risk of developing cognitive disorders and indicated the potential shared pathological mechanisms between the conditions.

Given the high prevalence of cognitive impairment in T2DM, early prediction is important for timely intervention and improved prognosis. Some studies reported compromised WM integrity in patients with T2DM prior to the onset of cognitive impairment [35] or in patients without MCI [49]. These impairments were associated with abnormal hippocampal functional connectivity, suggesting the possibility of early cognitive dysfunction [31]. These findings suggest that white-matter abnormalities may precede overt cognitive impairment in some individuals with T2DM.

#### 4.1.3. Findings in Clinical Subgroups

T2DM patients with concomitant obesity exhibited more severe abnormalities in brain structure and cognitive function compared to those without obesity [53]. Van Bloemendaal et al. found that obese T2DM patients had reduced WM integrity and volume in specific brain regions compared to lean controls, and that body mass index was an important factor affecting WM structure [51]. In a meta-analysis of eight studies, FA of the CC was found to be negatively correlated with body mass index in the patient cohort [59]. In patients with T2DM, body mass index correlated with localized diffusion homogeneity and FA value, in areas such as the right temporal lobe and left inferior parietal lobe, revealing that obesity may adversely affect WM microstructure [54].

Disease duration and WM microstructural changes in T2DM patients were positively correlated in most studies. Hsu et al. showed that MD values of brain parenchyma (cerebellum, temporal lobe WM, right caudate nucleus, etc.) were considerably higher in T2DM patients and were correlated with disease duration [29]. A study of T2DM patients with subjective memory complaints found that FA in the right superior longitudinal fasciculus was decreased and negatively correlated with disease duration, while Dr in the left posterior limb of the internal capsule was increased and positively correlated [38]. Serial DTI may provide a useful framework for investigating longitudinal microstructural changes. However, evidence supporting its role in monitoring disease progression or guiding individualized treatment remains limited.

Mood abnormalities, such as depression and anxiety, in patients with T2DM have also been associated with cerebral microstructural damage. A study by Roy et al. found that T2DM patients exhibited significantly increased MD in multiple brain regions (such as the cerebellum, insula and cingulate) [42]. Microstructural impairment in these regions was significantly associated with symptoms of depression or anxiety. Zhang et al. found that patients who had a comorbidity of depression and T2DM had a decreased FA in the anterior limb of the right internal capsule [45].

A study indicated that poor emotional memory may be more significant in female patients with T2DM compared to males, implying potential gender differences in WM microstructural abnormalities [44].

The interpretation of brain microstructural abnormalities in T2DM is complicated by multiple confounding factors. Therefore, current diffusion MRI findings should not be interpreted as purely diabetes-specific. Instead, they may reflect the combined effects of hyperglycemia, insulin resistance, obesity, and mood disorders.

#### 4.1.4. Association with Clinical Features

Glycemic control constitutes the cornerstone of T2DM management. Glycated hemoglobin (HbA1c) levels were found to be positively correlated with FA values in regions such as the CC and right hippocampus [32,60]. Hoogenboom et al. observed that fasting glucose was negatively correlated with FA values in the cingulate bundle in patients with T2DM [46]. Nouwen et al. found that HbA1c was negatively correlated with overall brain WM microstructural integrity [52]. However, there are also studies that have reached different conclusions. Raffield et al. found that after adjusting for a variety of factors, neither HbA1c nor the duration of diabetes was significantly associated with neuroimaging indices [47]. This discrepancy may be related to factors such as characteristics of the study population, sample size, and study methodology.

Liang et al. found that systolic blood pressure correlated with localized diffusion homogeneity and FA in the right temporal pole and bilateral orbitofrontal area, suggesting that hypertension affected the cerebral WM microstructure, and increased the risk of cognitive decline [54]. Cui et al. showed that insulin resistance was negatively correlated with FA values of bilateral lingual gyrus fibers [55]. van Bloemendaal et al. showed that fasting insulin levels, among others, correlated with WM integrity and volume [51]. Zhang et al. found that plasma Nε-(carboxymethyl)-lysine levels negatively correlated with whole-brain WM FA values in T2DM patients [50]. The association of these metabolic indices with DTI indices provided additional clues for a comprehensive understanding of the mechanisms of cerebral alterations.

### 4.2. Application of DTI-Based Network Analysis in T2DM

Topological characteristics of the structural network in T2DM patients were modified. Regarding global network properties, Kim et al. found that WM network efficiency was reduced (represented by decreased global efficiency and local efficiency and prolonged Lp) in T2DM patients, and HbA1c levels were negatively correlated with network efficiency and positively correlated with Lp. This suggests that a hyperglycemic state may impact the topological integration of brain network, leading to a decrease in the efficiency of information transfer [61].

The node properties of the structural network were also significantly altered in T2DM [62]. Li et al. reported that T2DM-MCI patients showed markedly compromised structural network at both global and regional levels, including the limbic system, basal ganglia, and multiple cortical regions, characterized by decreased nodal efficiency and reduced connectivity [15] (Figure 2B). In individuals with T2DM without complications, nodal efficiency decreased in the right hippocampus, right amygdala, and left pallidum, whereas nodal degree increased in the right inferior frontal gyrus [63]. Studies comparing cognitively impaired T2DM patients with healthy controls revealed reduced nodal efficiency across multiple brain regions. In contrast, comparisons between cognitively normal T2DM patients and healthy controls showed fewer regions with such reductions [14] (Figure 2C) (Table 3).

Without cognitive impairment, global network properties and the nodal efficiency of the right Rolandic operculum both had positive correlations with executive function [64]. Furthermore, the duration of diabetes mellitus was negatively correlated with global efficiency, positively correlated with clustering coefficient and Lp, and HbA1c level was correlated with Lp [14]. These results suggested that DTI-based network parameters were closely related to disease severity in T2DM.

### 4.3. Application of DKI in T2DM

In published DKI studies, besides decreased FA and increased MD, other kurtosis metrics provided additional information. The MK, axial kurtosis, and radial kurtosis values of the splenium of the CC and the pons in T2DM patients were significantly reduced [65]. In other studies, MK and radial extracellular spatial diffusivity could detect more regions of microstructural changes than FA and MD [39,66]. This suggested that MK may be more sensitive than DTI in detecting microstructural changes, possibly owing to its sensitivity in detecting changes in crossing fibers. Xie et al. revealed that microstructural abnormalities in the right prefrontal lobe deteriorated with the prolonged course of the disease. This indicates that the cerebral impairment due to T2DM progressively escalates with the progression of the disease [65]. In addition to WM, DKI has yielded significant discoveries in identifying microstructural alterations in gray matter. A study revealed that patients had greatly decreased MK values in the bilateral thalamus and caudate nucleus, suggesting a reduction in the complexity of grey matter structures [39] (Figure 3B).

MK values in the cingulate gyrus (hippocampus) were found to be positively correlated with MoCA and Mini Mental State Examination scores in patients with T2DM [39], while FA values in the right prefrontal WM negatively correlated with response time to the Attention Network Test [65], suggesting that these microstructural changes affected patients’ cognitive function.

### 4.4. Application of NODDI in T2DM

**Figure 4:**
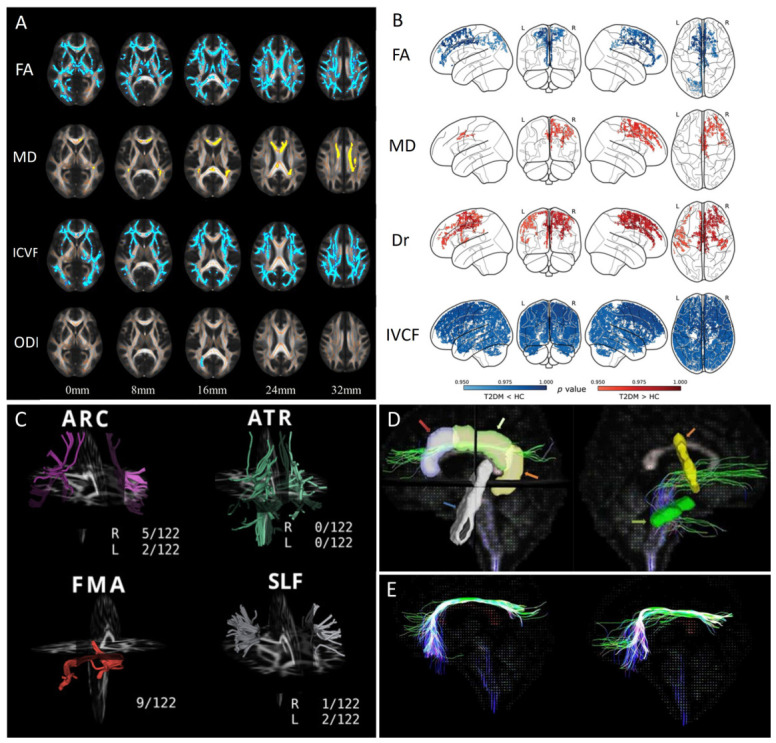
Application of diffusion models in type 2 diabetes (NODDI and DSI). Differences in FA, MD, and NODDI metrics between T2DM and controls. Blue: regions with decreased metrics. Yellow: regions with increased metrics (**A**). Patterns of the altered microstructure of the gray matter between T2DM and controls. Color bar represents the (1 − *p*) values (**B**). The number of subjects for which automatic fiber quantification tracking failed is noted below (**C**). ARC: arcuate fasciculus; ATR: anterior thalamic radiation; FMA: forceps major; SLF: superior longitudinal fasciculus. For superior cingulum bundle reconstruction, three ROIs were placed on the anterior, middle, and posterior cingulate gyri. For reconstruction of the uncinate fasciculus, one ROI was placed on an axial slice after curvature to the temporal lobe and the other on a coronal slice in the frontal lobe (**D**). Superior cingulum bundle tractography (**E**). Panel (**A**) was adapted with permission from Xiong et al. [67]. Panels (**B**–**E**) were adapted from the original publications [68,69,73], distributed under the terms of the Creative Commons Attribution License (CC BY).

Several investigations employing the NODDI model have identified substantial microstructural alterations in brain. Xiong et al. found that FA and ICVF were reduced in some WM regions in patients with T2DM. The changes were more extensive and obvious in T2DM patients with MCI, suggesting decreased density of axons and dendrites [67]. Studies observed reduced FA and ICVF and increased MD, Da, Dr and orientation dispersion index in T2DM participants compared to controls [68,69,70] (Figure 4A,B). In the study comparing DTI and NODDI, both analyses demonstrated that the hippocampus-amygdala transition area was a subregion that was particularly sensitive to microstructural changes in patients with T2DM [71].

The ICVF values in the genu of CC and thalamus were significantly correlated with HbA1c levels, disease duration, and neuropsychological test scores, suggesting that changes in neuronal synaptic densities in these regions are closely related to disease severity and cognitive function [67]. NODDI-based metrics showed potential group-level discrimination between T2DM patients and healthy controls in a limited number of studies [68].

### 4.5. Application of IVIM, DSI and FWI in T2DM

The IVIM model was able to sensitively detect microstructural and microvascular changes. An IVIM study found that elevated microvascular pseudo-diffusion coefficient and the diffusion coefficient value negatively correlated with decreased memory performance [72].

DSI was applied to assess the microstructural integrity of WM fibers by calculating the GFA [73]. The GFA values of the left uncinate fasciculus and the right superior cingulum bundle in patients with T2DM were significantly lower than those of the healthy controls, which indicated that significant degeneration had occurred in these fiber bundles. Huang et al. performed DSI on patients with T2DM, together with DTI and NODDI analyses, and found that microstructural changes existed in the right superior longitudinal fasciculus, right arcuate fasciculus, left anterior thalamic radiation, and forceps major [69]. The DSI parameters were associated with clinical indicators. Zhang et al. found that the GFA values of the right superior cingulum bundle in T2DM patients, which were negatively correlated with Verbal Fluency Test scores and inversely linked with total cholesterol levels (Figure 4C–E).

To the best of our knowledge, no studies have investigated the use of FWI in T2DM.

## 5. Discussion

Advanced dMRI models provide complementary approaches for investigating brain microstructural alterations associated with T2DM, although the strength of evidence varies substantially across techniques. Various dMRI techniques, including DTI, DKI, NODDI, IVIM, and DSI, provide multifaceted and complementary insights into T2DM-related brain alterations. These abnormalities are frequently associated with disease severity and cognitive impairment, particularly in memory, executive function, and information processing speed.

Among all diffusion MRI approaches, DTI provides the most consistent and reproducible findings. DTI has been widely used to assess WM integrity and has revealed widespread abnormalities in multiple brain regions, including the frontal, temporal, and parietal lobes, as well as major fiber tracts such as the CC and cingulate bundles. As summarized in Table 2 and illustrated in Figure 3, the most consistent DTI findings were reduced FA and increased diffusivity in long-range association fibers, interhemispheric pathways, limbic tracts, and corticothalamic/cerebellar circuits. In contrast, findings in smaller or region-specific tracts were less consistent and may depend more strongly on differences in study population, analytical strategy, and correction for vascular or metabolic covariates.

Evidence from DTI-based structural network analyses is moderately consistent. Beyond regional WM abnormalities, DTI-based network analyses further indicate that T2DM-related brain injury involves altered structural network organization. As shown in Table 3 and Figure 2, patients with T2DM, particularly those with MCI, frequently exhibit reduced global and nodal efficiency and increased characteristic path length, suggesting impaired network integration and less efficient information transfer. These network-level findings provide an important bridge between regional microstructural damage and clinically observed cognitive impairment.

In contrast, evidence from DKI, NODDI, IVIM, DSI, and FWI remains comparatively limited and should be regarded as emerging rather than established. While these techniques have shown promise in detecting microstructural abnormalities beyond those captured by conventional DTI, the available evidence is based on relatively few studies. DKI may provide additional sensitivity to microstructural alterations in both white and gray matter. Evidence from NODDI, IVIM, and DSI is more preliminary but suggests that these approaches may offer complementary information regarding neurite architecture, microvascular alterations, and complex fiber organization.

Most available studies are cross-sectional and report group-level differences rather than individual-level diagnostic or prognostic performance. Few studies have examined test–retest reliability, external validation, or longitudinal predictive value for cognitive decline. Diffusion MRI findings may be influenced by differences in scanner manufacturers, magnetic field strength (1.5T vs. 3T), diffusion acquisition schemes, number of diffusion-encoding directions, and b-values. In addition, preprocessing procedures, including motion correction, eddy-current correction, tensor fitting algorithms, tractography methods, and region-of-interest definitions, vary substantially among studies. Statistical approaches also differ, particularly with respect to adjustment for confounding variables and correction for multiple comparisons. These methodological differences may contribute to variability in the location, extent, and significance of reported microstructural abnormalities and likely explain some of the inconsistencies observed across studies.

Moreover, interpretation of diffusion MRI abnormalities in T2DM is further complicated by several clinical and metabolic factors reported across the included studies. Obesity, hypertension, insulin resistance, disease duration, glycemic control, and mood disorders have all been associated with alterations in diffusion metrics. Several studies reported correlations between HbA1c levels, blood pressure, disease duration, or insulin resistance and measures of white-matter integrity, suggesting that observed diffusion abnormalities may reflect the combined effects of metabolic and vascular dysfunction rather than diabetes alone. In addition, depression and anxiety symptoms were associated with microstructural alterations in several brain regions, further complicating interpretation of disease-specific effects. Future studies should systematically measure and adjust for these metabolic, vascular, and neuropsychiatric factors to better distinguish diabetes-related effects from broader contributors to brain microstructural alterations.

## 6. Conclusions

Differences in study design, patient populations, and imaging protocols across studies make it difficult to draw definitive conclusions. Future research should move beyond describing group-level diffusion abnormalities and focus on more mechanistically specific and clinically translatable approaches. First, advanced diffusion models that have not yet been fully explored in T2DM, such as FWI and more flexible NODDI-related or multi-compartment diffusion frameworks, should be applied to clarify whether diffusion abnormalities primarily reflect axonal injury, demyelination, neurite loss, extracellular free-water expansion, microvascular dysfunction, or a combination of these processes. In particular, FWI may help separate free-water-related changes from tissue-specific microstructural damage, whereas variable-diffusivity extensions and multi-kernel approaches may reduce parameter bias caused by fixed intrinsic diffusivity assumptions in conventional NODDI by allowing tissue response functions or intrinsic diffusivity parameters to be modeled more flexibly rather than being fixed a priori. Second, future studies should integrate dMRI with complementary neuroimaging modalities, including functional MRI, arterial spin labeling, susceptibility-sensitive imaging, and magnetic resonance spectroscopy, as well as metabolic and inflammatory biomarkers, to better characterize the interactions among hyperglycemia, cerebrovascular dysfunction, neuroinflammation, and cognitive decline. Third, machine learning and radiomics-based approaches could be used to combine multi-parametric diffusion features, clinical variables, cognitive measures, and metabolic biomarkers to develop predictive models for early cognitive impairment, risk stratification, and longitudinal monitoring in patients with T2DM. However, these models should be validated in large, multicenter, longitudinal cohorts with standardized acquisition protocols and external validation to ensure reproducibility and clinical applicability.

## Figures and Tables

**Table 1:** Comparative overview of diffusion MRI models for detecting T2DM-related brain microstructural alterations.

Model	Main Metrics	Sensitivity	Clinical Feasibility	Evidence Maturity in T2DM
DTI	FA, MD, Da, Dr	Moderate; sensitive to WM injury	High	Highest
DTI-network	Global/local/nodal efficiency, characteristic path length, etc.	Sensitive to network disruption	Moderate	Moderate
DKI	MK, Ka, Kr, DTI-derived diffusivity	Higher than DTI for non-Gaussian diffusion	Moderate to low	Moderate
NODDI	ICVF, ODI, ISOVF	Sensitive to neurite density/orientation changes	Moderate to low	Emerging
IVIM	D, D*, f	Sensitive to microstructure and microperfusion	Moderate	Limited
DSI	GFA, fiber orientation distribution	High for complex fiber architecture	Low	Limited
FWI	Free-water fraction	Potentially useful for separating extracellular water from tissue injury	Moderate	Not yet studied in T2DM

T2DM: type 2 diabetes mellitus; DTI: diffusion tensor imaging; DKI: diffusion kurtosis imaging; NODDI: neurite orientation dispersion and density imaging; IVIM: intravoxel incoherent motion; DSI: diffusion spectrum imaging; FWI: free-water imaging; FA: fractional anisotropy; MD: mean diffusivity; Da: axial diffusivity; Dr: radial diffusivity; WM, white matter; MK: mean kurtosis; Ka: axial kurtosis; Kr: radial kurtosis; ICVF: intracellular volume fraction; ODI: orientation dispersion index; ISOVF: isotropic volume fraction; D: diffusion coefficient; D*: pseudo-diffusion coefficient; f: perfusion fraction; GFA: generalized fractional anisotropy.

**Table 2:** An overview of DTI analyses in the review.

Study	Lower FA	Higher MD	Higher Da	Higher Dr
Yau et al. [44]	L-TS, R-EC R-prefrontal region L-frontal temporal region	\	\	\
Yau et al. [27]	L-TS, L-CP R- cingulate WM	R-STG, L-prefrontal cortex, R-parietal cortex	\	\
Hsu et al. [29]	FL	anterior and posterior lobes of cerebellum, L-PHG, TL, L-fusiform gyrus, L-cuneus	Same as MD	Same as MD
Reijmer et al. [36]	R-UF	SLF, UF, ILF, s CC	SLF, UF, L-ILF, s CC, trend in R-ILF	SLF, UF, L-ILF, trend in R-ILF
Falvey et al. [28]	Global WM	Hippocampus, L-PC, R-putamen, dorsolateral prefrontal cortex	\	\
Aifeng Zhang et al. [45]	T2DM with MDD: R-ALIC; trend in L-ALIC	No significant difference	No significant difference	T2DM with MDD: R-ALIC
Junying Zhang et al. [35]	TBSS: CC, CR, IC, PTR, L-CG, L-hippocampus, SLF, ILF, SFOF, EC, UF, IFOF, tapetum, fornix/stria terminalis	TBSS: CC, CR, EC, IC, SLF, PTR, tapetum, cingulum (cingulate gyrus)	No significant difference	TBSS: CC, CR, IC (except R-PLIC), PTR, CG, SLF, IFOF, EC, fornix/stria terminalis, UF, tapetum, R-SFOF
Hoogenboom et al. [46]	Cingulum bundle, UF	No significant difference	Trend in lower Da in cingulum bundle	No significant difference
Raffield et al. [47]	Lower FA in WM and GM	Higher MD in WM and GM	\	\
van Bussel et al. [48]	No significant difference	No significant difference	\	\
Tan et al. [30]	right cingulum-frontal lobe, cingulum-parietal lobe, vermis cerebella, thalami	Middle temporal gyrus, thalami	Thalamus, R-corona radiata	No significant difference
Xiong et al. [49]	ROI: T2DM-MCI < T2DM-NC: L-EC, L-ALIC, ACR, L-PTR, hippocampus; T2DM-NC < HC: R-CST and R-CP	ROI: T2DM-MCI > T2DM-NC: L-EC, L-RIC, L-SCR, R-SS; T2DM-NC > HC: R-RIC and R-EC	TBSS: T2DM-MCI > HC: several regions	TBSS: T2DM-NC > HC: EC, temporal WM, R-frontal WM and CR
Jian-Hui Zhang et al. [50]	CF, IFOF, CC, TL, hippocampus, parietal WM	\	\	\
van Bloemendaal et al. [51]	No significant difference	No significant difference	Obese T2DM < HC: R-CST, R-IFOF.R-SLF, R-F Ma; Obese without DM < HC: trend in L-F Ma	No significant difference
Fang et al. [43]	The cerebellar circuit; cerebro–cerebellar circuit	\	\	\
Nouwen et al. [52]	T2DM < HC: L-CST, medial CC, L-fornix, L-TR, L-RIC, L-IFOF, R-ACR, L-uncinate, L-CC, cingulum, L-AEC	No significant difference	No significant difference	\
Yoon et al. [53]	ROI: overweight-obese T2DM < normal-weight T2DM: prefrontoparietal WM	\	\	\
Sun et al. [31]	TBSS: CC, cingulum, CST, IC, EC, ACR, PCR, fornix, PTR, SCP, tapetum; ROI: g CC, b CC, L-CST, L-SCP, L-PTR	TBSS: same as FA; ROI: b CC, s CC, R-ACR, SCR, PCR, R-cingulum	TBSS: same as FA; ROI: b CC, s CC, R-ACR, SCR, PCR	TBSS: in restricted brain regions; ROI: b CC, s CC, R-ACR, SCR, PCR
Yu et al. [32]	In stroke-T2DM: I CC	No significant difference	No significant difference	I CC
Liang et al. [54]	L-SCR	\	\	\
Zhuo et al. [40]	T2DM with microvascular disease vs. NC: CC, ALIC, R-RIC, PTR, R-SLF, SCR and L-MFG;	T2DM with microvascular disease vs. NC: CC, ALIC, R-RIC, PTR, R-SLF, SCR and L-MFG;	\	\
Cui et al. [33]	Cingulum bundle	R-cingulum bundle	\	\
Wang et al. [38]	R-SLF, arcuate, R-CST	L-CST, IFOF, R-ILF, L-CC	L-CST, L-arcuate	L-TR
Cui et al. [55]	lingual gyrus	\	\	\
Roy et al. [42]	\	The cerebellum, insula, and frontal and prefrontal cortices, cingulate, LG	\	\
Liu et al. [37]	\	CF Ma, CF Mi, R-IFOF, R-ILF	CF Ma, R-IFOF, R-ILF, R-SLF	CF Ma, bilateral IFOF

R and L: right and left side; ACR: anterior corona radiata; AEC: anterior external capsule; ALIC: anterior limb of internal capsule; CC: corpus callosum; b CC: body of CC; g CC: genu of CC; I CC: isthmus of CC; s CC: splenium of the CC; CF: cingulate fasciculus; CF Ma: callosum forceps major; CF Mi: callosum forceps minor; CG: cingulate gyrus; CR: corona radiata; CST: corticospinal tract; CP: cerebral peduncle; EC: external capsule; HC: healthy controls; FL: frontal lobe; F Ma: forceps major; IC: internal capsule; IFOF: inferior fronto-occipital fasciculus; ILF: inferior longitudinal fasciculus; LG: lingual gyrus; MDD: major depressive disorder; MFG: middle frontal gyrus; PC: posterior cingulate; PCR: posterior coronal radiata; PHG: parahippocampal gyrus; PTR: posterior thalamic radiation; RIC: retrolenticular internal capsule; SCP: superior cerebellar peduncle; SCR: superior corona radiata; SFOF: superior fronto-occipital fasciculus; SLF: superior longitudinal fasciculus; SS: sagittal stratum; STG: superior temporal gyrus; TL: temporal lobe; TR: thalamic radiation; TS: temporal stem; UF: uncinate fasciculus; WM: white matter.

**Table 3:** An overview of DTI-based network analyses in the review.

Study Groups	Clustering Coefficient	Local Efficiency	Shortest Path Length	Global Efficiency	Strength	Nodal Efficiency	Small-Worldness
Reijmer et al. [36]	↓	→	↑	↓	\	\	\
Kim et al. [61]	→	\	↑	↓	→	\	→
Junying Zhang et al. [64]	→	↓	↑	↓	↓	↓	↓
Yang Zhang et al. [63]	↑	→	↑	→	\	↓	→
Vergoossen et al. [62]	Prediabetes vs. HC ↓; T2DM vs. HC →	Prediabetes vs. HC ↓; T2DM vs. HC →	\	→	\	\	\
Xiong et al. [14]	\	T2DM-MCI vs. HC ↓; T2DM-NC vs. HC →	T2DM-MCI vs. HC ↑; T2DM-NC vs. HC →	T2DM-MCI vs. HC ↓; T2DM-NC vs. HC →	\	T2DM-MCI vs. HC ↓; T2DM-NC vs. HC ↓	\
Li et al. [15]	→	T2DM-MCI vs. HC ↓; T2DM-NC vs. HC →	↑	↓	\	↓	→

An upward arrow indicates an ascending, a downward arrow indicating a descending, a rightward arrow indicating no significant change, and “\” indicating no mention.

## Data Availability

Data sharing is not applicable to this article as no new data were created or analyzed in this study.

## Supplementary Materials

The following supporting information can be downloaded at: https://www.mdpi.com/article/10.3390/bioengineering13070730/s1, Table S1. Methodological quality assessment of the included studies.

## Abbreviations

CC	corpus callosum
Da	axial diffusivity
DACD	diabetes-associated cognitive dysfunction
DKI	diffusion kurtosis imaging
dMRI	diffusion magnetic resonance imaging
Dr	radial diffusivity
DSI	diffusion spectrum imaging
DTI	diffusion tensor imaging
FA	fractional anisotropy
FWI	free-water imaging
GFA	generalized fractional anisotropy
HbA1c	glycated hemoglobin
ICVF	intracellular volume fraction
IVIM	intravoxel incoherent motion
Lp	characteristic path length
MCI	mild cognitive impairment
MD	mean diffusivity
MK	mean kurtosis
MoCA	Montreal Cognitive Assessment
NODDI	neurite orientation dispersion and density imaging
T2DM	type 2 diabetes mellitus
WM	white matter
